# A biokinetic model to assess radon uptake by the fetus during pregnancy

**DOI:** 10.1007/s00411-025-01187-3

**Published:** 2026-01-09

**Authors:** Ä. L. Degenhardt, V. Spielmann, A. Giussani

**Affiliations:** Division of Medical and Occupational Radiation Protection, Federal Office for Radiation Protection, Ingolstädter Landstraße 1, 85764 Oberschleißheim, Germany

**Keywords:** Radon, Internal dosimetry, Fetal dosimetry, Biokinetic modeling, Pregnancy, Radiation protection, Maternal-fetal transfer

## Abstract

Epidemiological studies have shown a statistically significant increase in lung cancer risk from prolonged exposure to indoor radon. While current radiation protection efforts address the general population, including workers in radon-prone areas, pregnant individuals represent a vulnerable subgroup that requires specific consideration. Prenatal exposure to ionizing radiation, including radon and its progeny, raises concerns not only for maternal health but also for potential in-utero tissue reactions and/or cancer development in other life stages in the offspring. This study aimed to develop a comprehensive biokinetic model for radon to evaluate fetal uptake following maternal exposure through inhalation. The model was based on the latest ICRP age- and sex-specific biokinetic model for radon, adapted to include pregnancy-specific compartments such as the uterus, placenta, arterial and venous cord blood, and key fetal organs (lungs, brain, kidneys, thyroid, bone surface, liver, and adipose). Transfer rates were calculated using Fick’s law of passive diffusion. Maternal and fetal physiological changes throughout pregnancy, including tissue masses and blood flows, were incorporated. Model simulations show that, despite radon gas being predominantly exhaled after inhalation, a fraction crosses the placenta, reaching fetal tissues—particularly those with higher fat content. Additionally, due to the chemical affinity of radon and fatty tissues, fetal adipose tissue receives a significant proportion of radon, resulting in the highest predicted uptake among fetal tissues. This biokinetic model provides an approach to estimate fetal uptake of radon from maternal exposures, supporting more accurate assessments for radiation protection of pregnant individuals and their developing fetuses.

**Supplementary Information:**

The online version contains supplementary material available at 10.1007/s00411-025-01187-3.

## Introduction

For decades, the major concern related to exposures to radon focused primarily on male miners inhaling radon and its radioactive decay products. However, epidemiological studies on residential exposure have demonstrated a statistically significant increase in lung cancer risk from prolonged indoor radon exposure (WHO,^2009^). Consequently, current concerns extend to both men and women working in offices and facilities located in radon-prone areas.

Pregnant individuals are a critical group in radiation protection, particularly concerning internal exposure to radionuclides in both occupational and residential exposures. This concern arises from the fetal susceptibility to postconceptional effects of ionizing radiation. Radionuclides can cross the placenta, exposing the fetus directly, and irradiation can also occur due to radionuclide activity in maternal organs (ICRP 2002a). Noble gases, when absorbed into the blood, characteristically tend to accumulate in tissues rich in fat (Sakoda et al. 2010). Consequently, maternal and fetal fatty tissues—such as adipose tissue and bone marrow—are of particular interest for radiological protection.

Early experimental studies by Bagg (1922) and Sikov and colleagues (1984, 1990) investigated the placental transfer of ²²²Rn and ⁸⁵Kr in animal models, demonstrating equilibrium between activities in maternal and fetal tissues. These results indicated that radon can cross the placenta and reach fetal organs. Sikov conducted additional animal studies on the contribution of maternal intakes to fetal doses for several radionuclides. Fractions of radionuclides present in the whole fetus at different times after the injection of 1 µCi into the mother’s blood at different gestation ages were published in a technical report (Sikov and Hiu, 1996).

Based on these and other animal studies, the ICRP introduced in Publication 88 (ICRP, 2002) fetal-to-maternal concentration ratios (C_F_:C_M_) for various radionuclides, including radon and its progeny. Based on this ratio, the total fetal activity can be estimated from the activity concentration in the maternal organ and the fetal mass. However, these ratios are assumed to remain constant throughout gestation, ignoring changes in maternal physiology and fetal development. Radon kinetics are influenced by perfusion, tissue volumes, and partition coefficients—factors not represented by these constant ratios. The placenta is also not treated as a transfer compartment, and it is therefore not possible to model how fetal uptake changes over time or with gestational stage using this approach.

No biokinetic model that quantifies radon distribution in pregnant women or its transfer to the fetus is currently available. Leggett et al. (2013) developed a physiologically based biokinetic framework for noble gases, and Samuels et al. (2023) extended this work to produce age- and sex-specific models for radon in non-pregnant individuals. While these models represent important advances, they do not incorporate pregnancy-specific physiology and do not provide a quantitative description of radon transfer to the fetus using a compartmental approach.

Pregnancy involves substantial physiological changes, including increased blood flow to the uterus and kidneys, growth of fat tissue, and the development of the placenta with its exchange to fetal circulation—factors not represented in existing models. A pregnancy-specific model is therefore needed to provide a foundation for assessing fetal radiation dose. Biokinetic modeling also supports the calculation of dose coefficients, which are crucial for estimating exposures in epidemiological studies on childhood cancers or low-dose radiation effects.

The aim of this study is to develop the first compartmental biokinetic model describing the transfer of inhaled ²²²Rn from mother to fetus that explicitly accounts for maternal and fetal physiology. The model builds on the age- and sex-specific structure of Samuels et al. (2023) by introducing pregnancy-specific compartments—including the uterus, placenta, cord blood, and fetal tissues—and incorporates physiological data from ICRP Publications 88 and 89. Characterizing radon gas kinetics is an essential precursor to modeling the behavior of its progeny, which is the greatest contributor to the organ doses (Kendall and Smith 2002; ICRP 2024). This work represents an initial step toward a comprehensive framework for evaluating fetal exposure to radon and its decay products.

## Methods

The most recent age- and sex-specific biokinetic model for radon (Samuels et al. 2023) was used for the maternal systemic model. In their model, the authors assumed that radon transfer rates within the organs follow the Fick’s first law of passive diffusion, similarly to the radon biokinetic model published by the International Commission on Radiological Protection (ICRP) in the Occupational Intakes of Radionuclides (OIR) Part III (ICRP 2017).

**Fig. 1:**
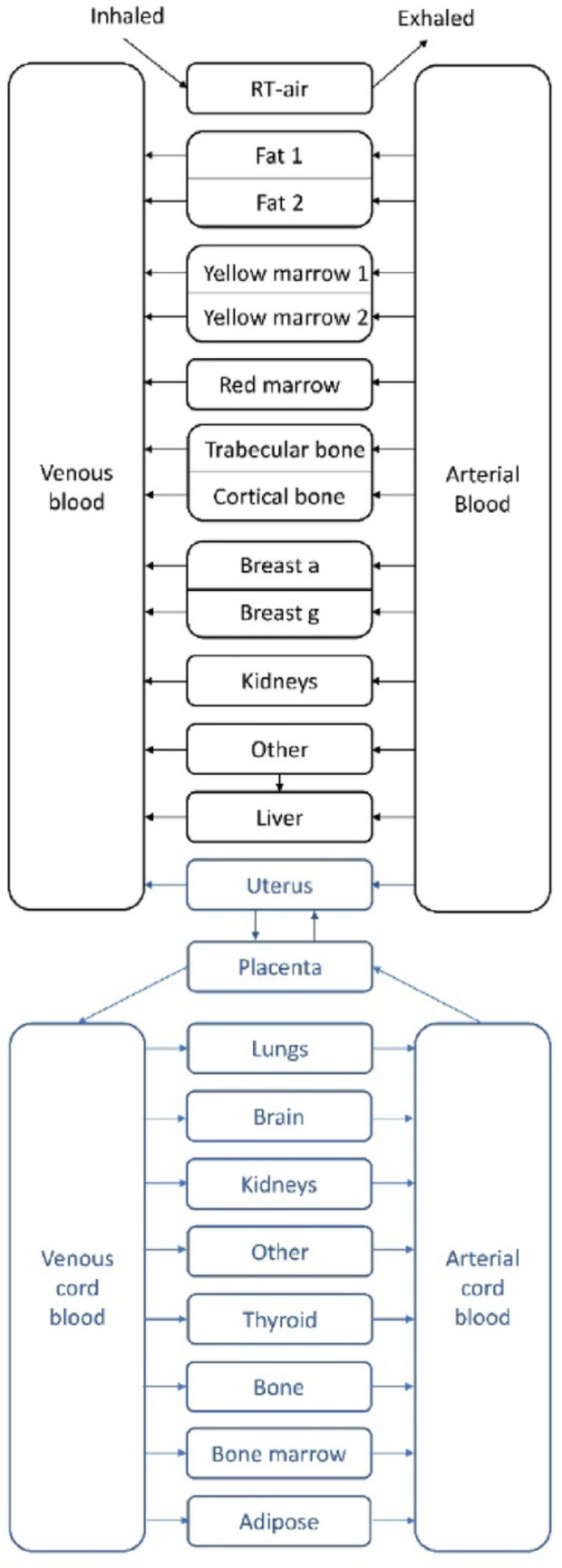
Proposed biokinetic model for inhalation of radon gas during pregnancy

To assess the fetal uptake of radon, the uterus was added to the adult systemic model and the fetal systemic model was created. The placenta was added to the maternal systemic model as an exchange compartment with the fetal systemic model. This includes compartments for the venous and arterial cord blood and some fetal organs relevant for radiation protection (i.e. thyroid, liver, bone surface, bone marrow, lungs, kidneys, and brain). Fetal adipose tissue was also accounted for given the fact that radon tends to accumulate in fat. Fetal adipose tissue is represented as a single compartment that includes all fetal fat, rather than multiple compartments as in the maternal model (Fat 1/Fat 2). In the model, radon that is transferred from maternal blood to the placenta is completely absorbed by the placenta and transferred to the fetal tissues through the venous cord blood. Radon is then washed out from the fetal tissues and goes back to the placenta through the arterial cord blood. Figure 1 below shows the proposed model for pregnancy with the new compartments depicted in blue.

The fetal organs considered in this biokinetic model—adipose, thyroid, brain, red bone marrow, bones, liver, lungs, and kidneys—were selected based on their known importance in radiation protection. These organs are critical due to their high radiosensitivity, essential roles in fetal development, and their relevance for estimating stochastic effects, including cancer risk following in utero exposure. This selection aligns with recommendations and modeling approaches established in ICRP Publication 88 (2001), which specifically identifies the brain, thyroid, and skeleton (bones – due to the presence of the bone marrow) for dose estimation in the fetus following maternal intake of radionuclides. Additional guidance from ICRP Publication 103 (2007) and UNSCEAR (2000) emphasizes the need to focus on these tissues due to their vulnerability during critical stages of fetal development.

### Physiological data for pregnancy and fetal development

According to the literature, the placenta is completely developed between the 12th and 15th week of pregnancy (84th – 105th gestation day) (Genbacev et al. 2003). For this reason, the proposed biokinetic model for the fetus can be applied from the 15th week of pregnancy (105th gestation day) to term. In the case of dose assessments for the pregnant individual, the model can be used from the first day of gestation.

During pregnancy, the body undergoes many changes. The typical mass gained during pregnancy amounts to 12.5 kg, according to ICRP Publication 89 (ICRP 2002b). Weight gain by the mother at different stages of pregnancy are given in Table 1. Changes in blood flow to selected tissues are given in Table 2.

**Table 1:** Weight gain during pregnancy (Table 12.2 ICRP 2002b adapted)

Tissues and fluids	Increase in mass (g) up to			
	10 weeks	20 weeks	30 weeks	38 weeks
Fetus	5	300	1500	3400
Placenta	20	170	430	650
Uterus	140	320	600	970
Breasts	45	180	360	405
Total	650	4000	8500	12,500

**Table 2:** Reference values for blood flow to selected organs of the non-pregnant and pregnant female near term (Table 2.44 ICRP 2002b adapted)

Organ/tissue	Blood flow rate (% of cardiac output)	
	Non-pregnant	Pregnant
Fat	8.5	7.8
Uterus	0.4	12.0
Breast	0.4	3.5
Other	3.2	3.3
Cardiac output (l/min)	5.9	7.3

As well as the maternal body is changing with the progress of the pregnancy, the fetus is changing during its development. The three phases of the development are (i) the pre-implantation period, (ii) the embryonic period of organogenesis, and (iii) the fetal period of organ growth. For dosimetric purposes, no distinction is made between phase (i) and (ii), which are both combined in the embryonic period. During this period, that comprises up to 8 weeks after the conception, the dose to the embryo is taken to be the same as the dose delivered to the uterus wall due to its small size (up to 10 g) (ICRP 2002a). During the early phase of the fetal phase (up to the 15th week of gestation) the absolute growth in fetal mass is relatively small. This phase is followed by a period when the major growth and changes in fetal organs and tissues happen, and it lasts up to 33–34 weeks after gestation (ICRP 2002a). In ICRP Publication 89, the accumulation of adipose tissue in the fetus is considered to begin around the 25th week of gestation (175th gestation day). Total fetal body fat is expressed as grams of lipid per 100 g of body mass, reflecting the increasing fat proportion as the fetus develops, specifically after the 25th week of gestation. The mass of fetal adipose tissue at each gestational age was estimated using lipid content values provided in Table 3 of ICRP Publication 89 (ICRP 2002b), combined with the corresponding fetal body masses also given in the same report. Based on these values, it was possible to calculate the absolute mass of adipose tissue from the 25th week of gestation onwards by applying the percentage of lipid content to the total fetal body mass. Table 3 (below) presents reference values for fetal organ masses at various gestational ages.

**Table 3:** Mass of selected fetal tissues at various times of development (Table 2.3 ICRP 2002a adapted)

Organ	Mass (g)						
	Week 8	Week 10	Week 15	Week 20	Week 25	Week 30	Week 38
Brain	3.7	6.4	22	59	118	192	352
Kidneys	0.022	0.12	1.2	3.5	7.0	12	23
Liver	0.19	0.81	6.1	17	35	59	120
Lungs	0.081	0.53	4.9	12.0	22	32	51
Red marrow	0.065	0.28	2.2	6.5	12	23	47
Bone surface	0.023	0.1	0.76	2.2	4.4	7.3	15
Thyroid	0.011	0.022	0.077	0.18	0.36	0.63	1.3
Total body	4.8	21	160	480	1000	1700	3500
Adipose	–	–	–	–	23.76	107.1	392

The ICRP has done an extensive work to provide reference values for pregnancy and fetus that can be applied in dosimetry (ICRP 2002b). However, reference values for fetal anatomy and physiology are not easily found in literature. The blood flow to some fetal organs were provided by Abjudalil et al. (2021), they carried out a literature search to collect and evaluate the changes that the fetal cardiac output and fetal tissue blood flows undergo during the development. The data collected were based on Doppler studies, blood flow through umbilical vein studies, ductus venosus studies, liver veins studies, brain studies, lungs studies and kidneys studies; and they allowed estimating physiologically based reference values. Table 4 shows the fetal blood flow (l/h) to some fetal organs at different gestation week presented by Abjudalil et al. (2021).

**Table 4:** Mean fetal blood flow (l/h) at different gestation weeks (Table 1 Abduljalil, 2021 adapted)

Organ blood flow (l/h)	Week 15	Week 22	Week 32	Week 40
Combined cardiac output	1.52	11.91	49.87	84.63
Umbilical vein	0.408	2.820	9.619	13.35
Liver	0.310	2.420	9.254	14.02
Brain	0.181	1.652	8.010	14.81
Lungs	0.336	2.620	10.97	18.62
Adipose	0.076	0.595	2.49	4.232
Kidneys	0.133	0.934	3.294	4.753
Bones	0.076	0.595	2.493	4.232

### Calculation of the transfer coefficients

**Fig. 2:**
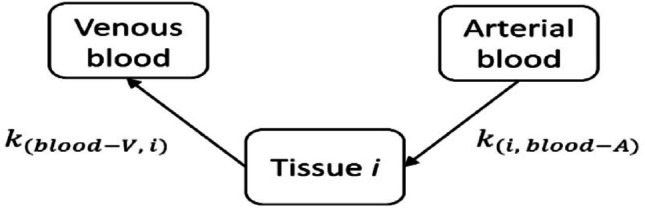
Three-compartment model composed by the arterial blood, the tissue *i*, and the venous blood

The transfer rates to and from the new compartments were calculated assuming that the transfer of radon among the organs follow the Fick’s first law of passive diffusion. Let’s consider a three-compartment model composed by the arterial blood, the tissue *i*, and the venous blood (Fig. 2).

Assuming that, for inhalation, the gas in the air space is taken to equilibrate with the blood in the lungs (Khursheed 2000), and that the perfusion of radon gas in the tissues is almost instantaneous, what allows the equilibrium between the gas in the blood and in the tissues, the transfer rate from arterial blood to the tissue *i* is given by:

1 $$\:{k}_{(i,blood-A)}=\frac{{F}_{i}}{{V}_{blood-A}}$$

being $\:{F}_{i}$ the blood flow to the tissue *i*, and $\:{V}_{blood-A}$ the volume of arterial blood.

The transfer from the tissue *i* back to blood (venous) is given by:

2 $$\:{k}_{(blood-V,i)}=\frac{{F}_{i}}{{V}_{i}{P}_{i\to\:Blood-V}}$$

being $\:{F}_{i}$ the blood flow to the tissue i, $\:{V}_{i}$ the volume of the tissue i, and $\:{P}_{i\to\:Blood-V}$ the partition coefficient from the tissue i to the venous blood.

Legget et al. (2013) listed tissue-to-blood partition coefficients for radon, xenon, krypton, and argon gathered from different in vivo and in vitro studies available in the literature. Tissue-to-blood partition coefficients for radon necessary to calculate the transfer coefficients from organs to venous blood are listed in Table 5 below.

**Table 5:** Estimated partition coefficients for radon for selected organs (Table 1 Leggett et al. 2013 adapted)

Tissue/Blood	Radon
Fat	11
Bone	0.4
Kidney	0.7
Liver	0.7
Brain	0.7
Lung	0.7

#### Uterus integration into the systemic model

The transfer of radon from arterial blood to the uterus was calculated considering pregnancy-specific adjustments to uterine blood flow and mass. Uterine blood flow was modeled as a function of gestation age, with values interpolated for specific time points (0, 70, 105, 140, 154, 175, 210, 224, and 280 gestation days, corresponding to 0, 10, 15, 20, 22, 25, 30, 32, and 40 weeks of gestation). The partition coefficient for the uterus was assumed to be equal to that of muscle tissue (0.4) (Leggett et al. 2013). The transfer from the uterus back to venous blood was determined based on uterine radon content, tissue-blood partitioning, and dynamic changes in tissue perfusion over time. These considerations ensured that both radon uptake and release by the uterus were realistically represented throughout pregnancy, reflecting the physiological increases in uterine mass and blood flow as gestation progresses.

#### Adjustments for fat and breast tissue

Similarly, interpolations were applied to fat and breast tissues for both mass and blood flow using the partition coefficients for radon provided in Table 5.

Following a typical convention used in radon biokinetic modeling, the breast tissue in the proposed model is divided into two distinct compartments: “Breast a” (adipose tissue) and “Breast g” (glandular tissue). This differentiation is important because radon has a higher solubility in fat compared to other tissue, resulting in greater retention in adipose tissue. Separating these compartments allows for more accurate estimation of radon uptake, retention, and dose distribution within breast tissue (Leggett et al. 2013).

In their biokinetic model for noble gases, Leggett et al. (2013) introduced two adipose compartments, “Fat 1” and “Fat 2,” each having equal volume but different blood flow (perfusion) rates. This distinction accounts for the varying retention behavior of radon in different fat tissues, reflecting differences in radon solubility and clearance related to blood perfusion. By differentiating these two types of fat, the model captures both rapid and long-term retention phases of radon in adipose tissue, enabling more precise estimation of radon storage and residence time across the body (Leggett et al. 2013).

#### Adjustments for kidney

During pregnancy, several physiological changes occur in the kidneys to support the increased demands of the body. Research shows that kidney mass can increase by up to 30%, primarily due to enhanced renal blood flow and structural adaptations such as increased vascular volume (Conrad 2004). According to ICRP Publication 89, pregnancy leads to significant increases in both glomerular filtration rate (GFR) and effective renal plasma flow (ERPF), with renal blood flow rising by about 70–80% by the beginning of the second trimester, followed by a slight decrease during the last trimester, but remaining 50–60% above pre-pregnancy levels at term (ICRP 2002b).

In our assumptions, we considered that by term, kidney mass increases by 30%, and we applied a linear interpolation for kidney mass values throughout gestation. For renal blood flow, we assumed an 80% increase by the second trimester – reaching this value at around the 15th week of gestation (105th gestation day), with a linear increase from the first trimester up to this point. Afterward, we modeled a linear decrease in blood flow from the 80% increase at the second trimester down to 50% above pre-pregnancy levels at term (40th week of gestation, 280th gestation day).

#### Placenta and uteroplacental circulation

The transfer of radon from the uterus to the placenta compartment in the model begins at the 15th week of gestation, consistent with the availability of fetal physiological data from this stage onward. Blood flow values from the uterus to the placenta were obtained from the study by Konje et al. (2001). Uterine blood flow increases from approximately 513 ml/min at the 20th week of gestation, and reaches around 970 ml/min by week 38. Given the lack of direct experimental measurements at the 15th week of gestation, the uteroplacental blood flow was assumed to be 350 mL/min. This assumption is consistent with the known near-linear increase in uteroplacental blood flow throughout pregnancy as described by Gude et al. (2004). For gestational ages not explicitly reported, blood flow values were estimated by interpolation, allowing for a continuous representation of uteroplacental blood flow throughout pregnancy.

The blood flow returning from the placenta to the uterus was assumed to be equal to the flow from the uterus to the placenta. This assumption reflects a closed and balanced uteroplacental circulatory system, where arterial inflow is matched by venous outflow. This is supported by Maltepe et al. (2022), who applied the Fick principle in ruminants and demonstrated that arterial blood flow to the placenta is balanced by venous return flow. Additionally, the study by Schmitt et al. (1984) on the ^133^Xe clearance method for placental blood flow utilizes a compartmental model of placental blood flow that inherently assumes a balanced, closed uteroplacental circulatory system, suggesting that maternal arterial inflow to the placenta is approximately equal to venous outflow.

The transfer coefficients for radon from the uterus to the placenta and from the placenta back to the uterus were calculated based on the blood flow values and organ volume. Applying these flow rates to Eq. 1 the transfer coefficients were then estimated representing the bidirectional exchange of radon between these compartments.

#### Fetal circulation

The model considers radon transfer from the placenta to the fetal circulation through the umbilical vein (UV), with the umbilical vein flow increasing dynamically from approximately 50 ml/min at 20th week of gestation (140th gestation day) to about 275 mL/min at term, following values reported by Bellotti et al. (2000). Regarding the blood flow from fetus to placenta, many studies report that approximately 30–40% of the fetal cardiac output is directed to the placenta via the umbilical arteries in humans, a range that is widely accepted (Kiserud et al. 2006).

#### Fetal tissue dynamics and blood flow

Fetal development was segmented into the embryonic period (up to 8 weeks) and the fetal period (ICRP 2002b), with maternal uterine dose considered as representative of fetal exposure before organogenesis. Growth and blood flow data were interpolated based on values reported by ICRP (2002a, b) and Abduljalil et al. (2021) (Tables 3 and 4). Additionally, fetal blood volume was adopted from Acharya et al. (2016), estimated as 16.2 ml per 100 g of fetal mass.

#### Fetal transfer coefficients

The transfer of radon from arterial blood to lungs, brain, kidneys, bone, liver, and adipose were calculated using Eq. 1, while the transfer from those tissues to venous blood was determined using Eq. 2.

Due to the limited availability of data on tissue-to-blood partition coefficients for fetal tissues, these coefficients were assumed to be equivalent to those determined for adult tissues. This assumption was necessary to allow modeling and analysis in the absence of fetal-specific partitioning information, recognizing that physiological differences may exist but currently lack quantitative characterization. The partition coefficients for fetal tissues, necessary for these calculations, were adopted to be the same as for an adult (Table 5).

For tissues lacking specific flow data (thyroid and red bone marrow), transfer coefficients were scaled based on the “other” tissue compartment. This compartment is defined as the total fetal mass minus the sum of all explicitly modeled tissues. Importantly, the transfer of radon to and from the “other” compartment, along with tissues such as the thyroid and red bone marrow (which are modeled as mass fractions of the “other” compartment), are only considered in the model from the 16th week of gestation (126th gestation day). Their transfer coefficients were linearly interpolated from zero at the 126th gestation day to their full values at the 140th gestation day, ensuring a gradual physiological growth of the organ rather than an abrupt appearance. Similarly, the transfer of radon to the adipose tissue is introduced at the 23th week of gestation (161th gestation day), with its transfer coefficient linearly interpolated from zero at the 161 st gestation day to its full value at the 175th gestation day. This gradual inclusion of compartments prevents unrealistic sudden changes in tissue mass and transfer properties.

## Results

### Transfer coefficients in maternal and fetal compartments

As described in Sect. 2.2.1 to 2.2.3, the transfer coefficients from arterial blood to maternal tissues, particularly the uterus, breast, and fat compartments, were calculated using Eq. 1 based on tissue-specific blood flow and volume data. These calculations account for the dynamic physiological changes that occur throughout pregnancy, including variations in tissue mass and blood flow, as established in Sect. 2.

The impact of these physiological changes in the mass and blood flow to maternal kidneys throughout pregnancy varies across gestation. In the second trimester (105th day of gestation – 15th week), the transfer coefficient was approximately 196% higher than those for a non-pregnant adult female, while at term they remained around 186% higher (280th day of gestation – 40th week). This highlights the importance of incorporating gestation-specific adjustments for accurate modeling of renal transfer dynamics during pregnancy.

The transfer coefficients between arterial blood and the compartment labeled “other,” as well as from “other” to venous blood, were also affected by the changes in the model. Since the “other” compartment represents a group of organs that are not individually defined, and we have now explicitly included the uterus as separate compartment in the maternal systemic model, its blood flow and mass contribution must be removed from the “other” group. This means that the transfer coefficients for uterus are subtracted from the original “other” compartment values to avoid double-counting. The new value of the transfer coefficient from the arterial blood to other is 5.02 × 10^3^ (day^−1^). Because the changes in the maternal tissues are explicitly handled separately for each relevant organ (fat 1 and 2, breast a and g, uterus, and kidneys), which undergo the most significant variations, no additional adjustments to the ‘other’ compartment are necessary during pregnancy, and therefore its transfer coefficient remains constant. The transfer coefficient from other to liver and other to venous blood remain the same as provided by Samuels et al. (2023).

For detailed values, please refer to the supplementary data section, where Supplementary Tables 1–6 provide the transfer coefficients (day^−1^) from the arterial blood to the different maternal tissues (uterus, Fat 1, Fat 2, Breast g, Breast a, and kidneys) and from those maternal tissues to the venous blood.

### Transfer rates in placenta, fetal circulation components, and some fetal organs

Supplementary Table 7 shows the transfer coefficients (day^−1^) for the exchange between uterus and placenta; Supplementary Table 8 shows the transfer coefficients from the placenta to the venous cord blood and from arterial cord blood to the placenta.

As explained earlier, after being transferred to the fetal circulation through venous cord blood, radon is distributed to the organs. The value for adipose remain constant throughout the fetal development and it is equal to 1.0 × 10^3^ (day^−1^). Although both the mass of fetal adipose tissue and its blood flow change during fetal development, the calculated transfer coefficient remains constant, likely due to the mathematical relationship between these parameters balancing out during the calculation. For detailed values, please refer to the supplementary data section, where the transfer coefficients from venous cord blood to some fetal organs are shown in the Supplementary Tables 9 and the transfer coefficients from organs to the arterial cord blood are shown in the Supplementary Table 10.

### Handling of limited data in fetal tissues

For fetal tissues such as the bone marrow and thyroid, where insufficient data precluded the direct use of Eqs. (1) and (2), transfer rates were estimated using a mass-ratio approach. This was achieved by calculating mass ratios relative to the “other” fetal compartment, at each of the key gestation ages (105^th,^ 140th, 154th, 175th, 210th, 224th, and 280th gestation days). This approach enabled consistent modeling of transfer dynamics even for compartments with limited direct physiological data.

### Time-dependency of transfer coefficients

Because of the progressive changes in both tissue masses and blood flows across gestation, the transfer coefficients calculated for maternal tissues (e.g., uterus, breast, fat) and fetal tissues (e.g., brain, lungs, kidneys) were found to be time-dependent. To model this temporal variability, the transfer coefficients computed at specific gestation ages were plotted in Microsoft Excel, and their change over time was described using linear regression equations of the form *k = a × gestational age + b*. These resulting functions allow for a continuous time-dependent description of transfer coefficients throughout pregnancy.

They are summarized in tabular form in Table 6 for maternal tissues and Table 7 for the placenta, fetal circulation components, and selected fetal organs - applicable only after the 105th day of gestation. Each table row indicates from which organs and to which organs the material is transferred, along with the corresponding coefficients *a* and *b*.

**Table 6:** Regression coefficients for modelling the time-dependency of transfer coefficients for maternal tissues

From	To	Gestation age (days)	a	b
Arterial blood	Uterus	8.6–280	3.99E + 00	−3.48E + 00
Uterus	Venous blood	A_g_ < 68.7	6.36E + 01	1.12E + 03
		A_g_ > 68.7	−1.03E + 01	6.19E + 03
Arterial blood	Fat 1	0–280	2.54E-01	5.27E + 02
Fat 1	Venous blood	0–280	−5.90E-03	5.93E + 00
Arterial blood	Fat 2	0–280	6.34E-02	1.32E + 02
Fat 2	Venous blood	0–280	−1.50E-03	1.48E + 00
Arterial blood	Breast g	0–280	7.38E-01	1.03E + 01
Breast g	Venous blood	0–280	1.44E + 00	1.09E + 02
Arterial blood	Breast a	0–280	4.43E-01	6.20E + 00
Breast a	Venous blood	0–280	8.10E-02	6.15E + 00
Arterial blood	Kidneys	0–70	4.41E + 00	1.33E + 03
		70–105	3.38E + 01	−9.75E + 02
		105–154	2.00E + 00	2.36E + 03
		154–210	−6.28E + 00	3.64E + 03
		210–280	1.67E + 00	1.97E + 03
Kidneys	Venous blood	0–70	−1.74E + 00	8.36E + 03
		70–105	1.85E + 02	−4.74E + 03
		105–154	−2.61E + 00	1.50E + 04
		154–210	−4.54E + 01	2.16E + 04
		210–280	−1.76E + 00	1.24E + 04

**Table 7:** Regression coefficients for modelling the time-dependency of transfer coefficients for fetal tissues

From	To	Gestation age (days)	a	b
Uterus	Placenta	105–160.7	−2.10E + 01	6.06E + 03
		160.7–210	6.11E + 00	1.70E + 03
		210–224	−7.13E + 01	1.80E + 04
		224–280	3.43E + 00	1.21E + 03
Placenta	Uterus	105–280	−1.95E + 01	7.19E + 03
	Venous cord blood	105–149.1	−9.61E-01	5.84E + 02
		149.1–280	2.18E + 00	1.15E + 02
Arterial cord blood	Placenta	105–197.9	3.60E + 01	−1.02E + 03
		197.9–280	−1.77E + 00	6.45E + 03
Venous cord blood	Lungs	105–198	2.63E + 01	−7.32E + 02
		198–280	−1.29E + 00	4.73E + 03
	Brain	105–280	1.43E + 01	−7.25E + 01
	Kidneys	105–198.5	6.26E + 00	1.64E + 02
		198.5–280	−3.55E + 00	2.11E + 03
	Thyroid	126–140	1.31E-02	−1.65E + 00
		140–280	7.00E-03	−7.96E-01
	Bone	105–206.5	7.83E + 00	−4.01E + 02
		206.5–280	−2.99E + 00	1.83E + 03
	Bone Marrow	126–140	4.65E-01	−5.85E + 01
		140–280	2.71E-01	−3.14E + 01
	Liver	105–280	1.23E + 01	1.02E + 03
	Other	126–140	4.79E + 01	−6.04E + 03
		140–280	1.62E + 01	−1.59E + 03
	Adipose	126–140	7.14E + 01	−1.15E + 04
Lungs	Arterial cord blood	105–280	6.14E + 01	3.11E + 03
Brain		105–280	7.16E + 00	−3.13E + 02
Kidneys		105–197.4	4.12E + 01	4.45E + 02
		197.4–280	−1.49E + 01	1.15E + 04
Adipose		161–175	7.11E + 00	−1.15E + 03
		175–220.4	−1.49E + 00	3.60E + 02
		220.4–280	−1.62E-01	6.78E + 01
Thyroid		126–140	2.90E-03	−3.65E-01
		140–280	1.20E-03	−1.27E-01
Bone		105–207.3	2.30E + 02	−1.37E + 04
		207.3–280	−8.29E + 01	5.12E + 04
Bone marrow		126–140	9.35E-02	−1.18E + 01
		140–280	4.64E-02	−5.19E + 00
Liver		105–198.9	2.07E + 01	−2.27E + 02
		198.9–280	−7.37E + 00	5.36E + 03
Other		126–140	3.47E + 00	−4.38E + 02
		140–280	2.67E + 00	−3.25E + 02

**Fig. 3:**
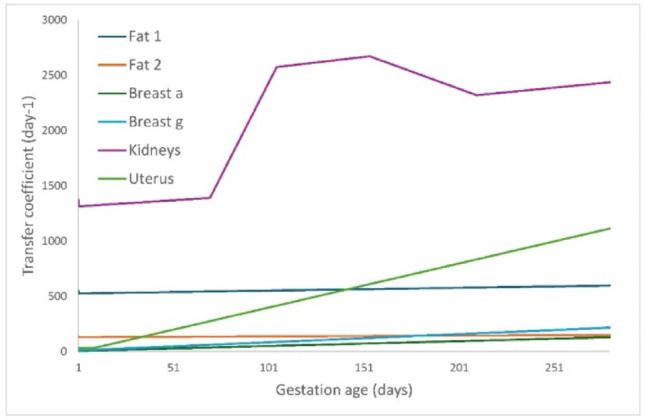
Changes in transfer coefficients from maternal arterial blood to maternal tissues

**Fig. 4:**
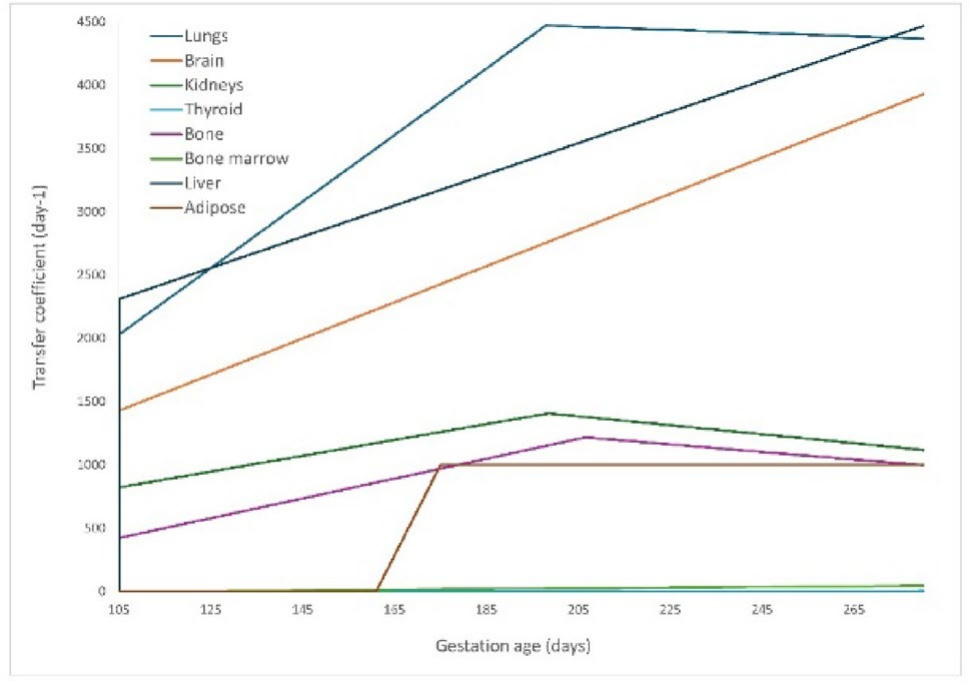
Changes in transfer coefficients from venous cord blood to fetal tissues

Figures 3 and 4 illustrate the variation in transfer coefficients over gestation. Figure 3 shows the changes in maternal arterial blood-to-tissue transfer coefficients for selected tissues, including the uterus, Breast a and g, and Fat 1 and 2, and kidneys as pregnancy progresses. Figure 4 depicts the fetal venous cord blood-to-organ transfer coefficients for fetal tissues demonstrating the influence of fetal growth and circulatory adaptations on radon transport dynamics. These figures highlight the radon transfer across compartments as pregnancy advances, driven by physiological and anatomical changes that were included in the modeling assumptions.

After solving the biokinetic model, we observed that for acute exposures, only up to 0.001% of the intake reaches fetal tissues. The short half-life of radon limits its transfer to fetal tissues, as much of the radon may decay before reaching them. While the direct transfer of radon to fetal tissues is limited, the contribution of radon progeny must also be considered, as these decay products may have longer retention times and present additional radiation exposure risks. Details on the biokinetics and dose coefficients for the full radon decay chain will be presented in a forthcoming publication.

**Fig. 5:**
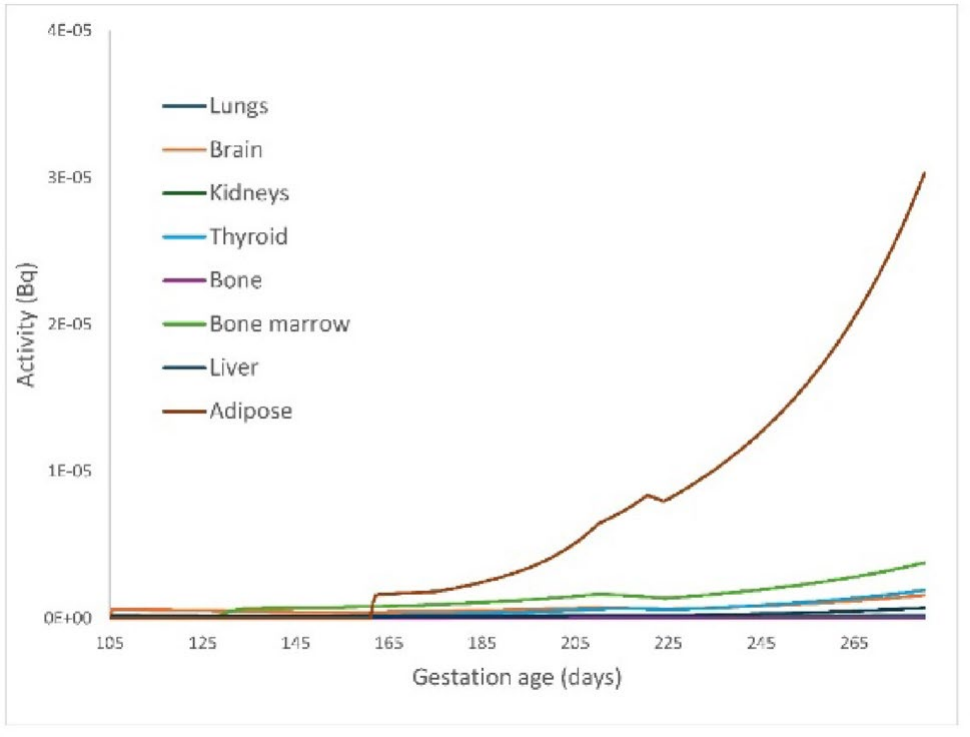
Content of radon in fetal tissues after chronic intake during pregnancy

In contrast, chronic exposure to radon—more common among individuals living or working in radon-prone areas – can lead to a gradual increase in radon activity within fetal tissues throughout pregnancy. During such chronic exposure scenarios, the fetal adipose tissue accumulates the highest radon concentration, followed by fetal bone marrow (Fig. 5). This aligns with fetal development patterns, as adipose tissue begins to accumulate significantly from the 25th week of gestation, with a marked increase in fat deposition during the third trimester. Although the transfer coefficient from fetal blood to adipose tissue is relatively low, the slow washout rate from this compartment, due to radon’s high solubility in fatty tissues, results in prolonged retention. Additionally, radon accumulation in fetal bone marrow warrants attention because of the potential ingrowth of alpha-emitting radon progeny. The model predictions shown in Fig. 5 correspond to an infusion of 1 Bq of radon administered daily from day 0 to day 280 of gestation.

## Discussion

Pregnancy induces substantial changes in radon transfer dynamics, mainly caused by increasing blood flow and tissue mass variations in specific maternal and fetal compartments. As gestation progresses, maternal tissues directly involved in offspring support (uterus, placenta, and breast) show increases in transfer coefficients, reflecting the physiological adaptations necessary to sustain fetal growth and prepare for breastfeeding. These findings are consistent with known pregnancy-related changes in cardiac output distribution, which prioritize uteroplacental circulation as gestation advances (Abduljalil et al., 2021).

In contrast, the transfer coefficients for fat compartments (Fat 1 and Fat 2) remained relatively stable throughout pregnancy, which can be explained by the fact that adipose tissue blood flow does not increase as drastically as in other maternal tissues (ICRP 2002b). Fat tissues are characterized by lower perfusion rates and slower exchange processes, making them less sensitive to hemodynamic changes during gestation. Furthermore, since radon has a strong chemical affinity for fat-rich tissues, its concentration in these tissues is driven more by solubility than by dynamic blood flow. This behavior is accounted for in the model through the application of partition coefficients, which determine the distribution of radon based on tissue solubility.

The dynamic changes observed in some maternal and fetal organs, emphasizes the importance of considering pregnancy-specific physiological adaptations in biokinetic modeling. The model outcomes also reinforce that fetal uptake of radon is significantly influenced by the increased placental and uterine blood flows, which enhance radon delivery to the developing fetus as pregnancy progresses.

### Uncertainties and limitations

It is important to recognize that the development of biokinetic models for pregnancy faces significant challenges due to the limited availability of physiological and anatomical data for the fetus, particularly regarding reference values for fetal tissue masses, blood flow distributions, and partition coefficients for specific radionuclides. Therefore, in this model, we have adopted an oversimplified representation of fetal circulation, drawing upon the work of Abduljalil et al. (2021), Acharya et al.,(2016), Bellotti et al. (2000), and Kiserud (2005).

While maternal physiological changes during pregnancy are relatively well-documented, data on fetal parameters—especially for late gestation stages—remain scarce, requiring the use of interpolations or assumptions based on limited datasets. This limitation affects not only radon modeling but biokinetic modeling in general. Therefore, more research is needed to provide accurate reference data for fetal tissues, enabling refinement of current models and improving the accuracy of dose estimates.

Direct experimental validation of radon transfer in human pregnancy is not currently possible because of ethical and practical limits. Even for similar noble gases such as xenon that have been applied in medical exposures, pregnancy-specific human data are very limited, since nuclear medicine procedures are rarely carried out during pregnancy and only in exceptional medical situations. As a result, there are no direct measurements of radon in the human fetus available for comparison.

A notable challenge in the model is the assumption that partition coefficients for radon in fetal tissues are equivalent to those in maternal tissues. This assumption stems from the lack of specific data on fetal tissue partition coefficients. Most existing data are derived from cell culture, animal studies, and simulations involving passive diffusion of noble gases in various mediums. Partition coefficients control how radon is transfer between the blood and the tissues, and therefore directly affect how much radon enters and stays in the tissues. If the true fetal partition coefficients are higher than the adult values used in this model, radon would be held longer in fetal tissues, leading to higher predicted concentrations. In contrast, if the fetal partition coefficients are lower, radon would remain mainly in the blood, resulting in lower tissue concentrations and faster return of radon to the placenta. These uncertainties mainly affect the level and the time radon remains in fetal tissues.

Uncertainty also comes from how fetal organ growth and blood flow were represented in the model. Fetal organ masses were taken from ICRP Publications 88 and 89 (ICRP 2002a, b), which provide reference values based on realistic growth models; and the blood flow values were obtained from Doppler studies in pregnant individuals (Abduljalil et al. 2021). These data are available only at specific gestational ages (masses for 10, 20, 30, and 38 weeks, corresponding to the gestation days 70th, 140th, 210th, and term; and blood flows for 15, 22, 32, and 40 weeks, corresponding to the gestation days 105th, 154th, 224th, and term). In this study, linear interpolation was used only between these adjacent ages, rather than extrapolating beyond the available data. Because the reference data are close enough to keep the main growth trends, the choice of interpolation method is not expected to introduce large additional uncertainties.

The use of Fick’s law of diffusion in a steady-state form implies that concentration differences across membranes adjust very quickly compared with changes in blood concentrations. In reality, diffusion may be slower under some conditions, especially when exposure changes rapidly. If these short-term effects were included, peak concentrations might be slightly higher or lower than those predicted by the model. However, because radon is an inert gas and the physiological processes in the maternal–fetal system generally occur over relatively slow time scales, the steady-state diffusion assumption is consistent with previous noble gas biokinetic models.

These assumptions and simplifications highlight the need for more comprehensive and specific data to refine biokinetic models for radon exposure during pregnancy.

### Radiological relevance and future developments

Although the model was built on a set of assumptions and simplifications of complex processes, it remains reliable and coherent based on the data currently available. It provides a novel tool for radiation protection purposes. While the direct uptake of radon gas by the fetus is very small, the principal aim of the work was to develop a compartmental structure, which can be used as a foundation for modeling the full chain of radon decay products. These progeny—²¹⁸Po, ²¹⁴Pb, and ²¹⁴Bi—are responsible for almost the entire radiation dose, often several orders of magnitude higher than that from radon gas itself due to the emission of alpha particles (Kendall and Smith 2002). Because progeny formation and retention depend on the local availability of radon in maternal and fetal tissues, accurately description of the kinetics of the parent is a necessary first step. With the description of the parent kinetics, the model allows future simulations of radon progeny transfer and accumulation, supporting the estimation of fetal doses in both occupational and residential exposure scenarios. A follow-up publication will present the progeny models and the resulting dose coefficients. It therefore represents an important step toward improving risk assessment and dose estimation in internal dosimetry studies, particularly for sensitive populations such as pregnant individuals.

## Conclusions

This study proposed a specific biokinetic model for radon in pregnancy. The dynamic changes in maternal physiology during pregnancy, including increased blood flow to the uterus, are crucial in enhancing radon delivery to the fetus. Furthermore, the particular characteristics of noble gases to accumulate in fat-rich tissues lead to high radon concentration in fetal adipose tissue. Our findings show that, while direct transfer of radon to fetal tissues is minimal, the accumulation of radon progeny, especially in tissues like the fetal adipose tissue, liver and red bone marrow, still could play a significant role for radiation risks.

The study also points out the challenges in developing accurate biokinetic models for pregnancy, due to the limited availability of data on fetal physiological parameters. There is a critical need for improving reference data for fetal tissues to refine current models and enhance the accuracy of dose estimations.

In conclusion, this work contributes to an understanding of radon uptake by the fetus during pregnancy. Developing more precise pregnancy-specific biokinetic models is essential for ensuring the safety of expectant mothers and their developing fetuses in radon-prone environments.

Future steps will include the development of pregnancy-specific biokinetic models for radon progeny (²¹⁸Po, ²¹⁴Pb, and ²¹⁴Bi), which contribute significantly to the radiation dose due to their alpha emissions and longer retention times compared to radon gas. Additionally, the calculation of dose coefficients for both maternal and fetal tissues should follow, based on the biokinetic models developed for radon and its progeny. These dose coefficients would provide a critical tool for risk assessment and regulatory purposes, offering a means to estimate fetal doses in various exposure scenarios, including chronic residential exposure and occupational settings.

## Supplementary Information

Below is the link to the electronic supplementary material.

Supplementary Material 1

## Data Availability

No datasets were generated or analysed during the current study.
