# Supplementary material for: A biokinetic model to assess radon uptake by the fetus during pregnancy
Source: Radiat Environ Biophys. 2026 Jan 9;65(1):135–45. doi: 10.1007/s00411-025-01187-3 (PMC13135538; doi:10.1007/s00411-025-01187-3)
Supplement: Supplementary file 1 — Supplementary Material 1 [file 411_2025_1187_MOESM1_ESM.docx]

**Supplementary Data**

Supplementary Table 1 - Transfer coefficients for Uterus (d^-1^)

| Gestation age (days) | Blood A to uterus | Uterus to blood V |
| --- | --- | --- |
| 0 | 3.09E+01 | 1.12E+03 |
| 70 | 2.70E+02 | 5.57E+03 |
| 105 | 4.00E+02 | 5.02E+03 |
| 140 | 5.36E+02 | 4.83E+03 |
| 154 | 5.92E+02 | 4.55E+03 |
| 210 | 8.28E+02 | 3.98E+03 |
| 224 | 8.90E+02 | 3.81E+03 |
| 280 | 1.15E+03 | 3.41E+03 |

Supplementary Table 2 - Transfer coefficients for Fat 1 (d^-1^)

| Gestation age (days) | Blood A to fat1 | Fat 1 to Blood V |
| --- | --- | --- |
| 0 | 5.25E+02 | 5.83E+00 |
| 70 | 5.45E+02 | 5.82E+00 |
| 105 | 5.54E+02 | 5.38E+00 |
| 140 | 5.63E+02 | 5.01E+00 |
| 154 | 5.67E+02 | 4.90E+00 |
| 210 | 5.80E+02 | 4.50E+00 |
| 224 | 5.84E+02 | 4.51E+00 |
| 280 | 5.96E+02 | 4.53E+00 |

Supplementary Table 3 – Transfer coefficients for Fat 2 (d^-1^)

| Gestation age (days) | Blood A to fat2 | Fat2 to Blood V |
| --- | --- | --- |
| 0 | 1.31E+02 | 1.46E+00 |
| 70 | 1.36E+02 | 1.46E+00 |
| 105 | 1.39E+02 | 1.35E+00 |
| 140 | 1.41E+02 | 1.25E+00 |
| 154 | 1.42E+02 | 1.22E+00 |
| 210 | 1.45E+02 | 1.12E+00 |
| 224 | 1.46E+02 | 1.13E+00 |
| 280 | 1.49E+02 | 1.13E+00 |

Supplementary Table 4 – Transfer coefficients for Breast g (d^-1^)

| Gestation age (days) | Blood A to Breast g | Breast g to Blood V |
| --- | --- | --- |
| 0 | 1.93E+01 | 7.97E+01 |
| 70 | 6.01E+01 | 2.27E+02 |
| 105 | 8.21E+01 | 2.77E+02 |
| 140 | 1.05E+02 | 3.19E+02 |
| 154 | 1.15E+02 | 3.31E+02 |
| 210 | 1.55E+02 | 3.72E+02 |
| 224 | 2.10E+02 | 4.98E+02 |
| 280 | 2.09E+02 | 4.76E+02 |

Supplementary Table 5 – Transfer coefficients for Breast a (d^-1^)

| Gestation age (days) | Blood A to Breast a | Breast a to Blood V |
| --- | --- | --- |
| 0 | 1.16E+01 | 4.48E+00 |
| 70 | 3.61E+01 | 1.28E+01 |
| 105 | 4.93E+01 | 1.55E+01 |
| 140 | 6.32E+01 | 1.80E+01 |
| 154 | 6.89E+01 | 1.86E+01 |
| 210 | 9.30E+01 | 2.09E+01 |
| 224 | 1.26E+02 | 2.80E+01 |
| 280 | 1.25E+02 | 2.68E+01 |

Supplementary Table 6 - Transfer coefficients for Kidneys (d^-1^)

| Gestation age (days) | Blood A to kidneys | Kidneys to Blood V |
| --- | --- | --- |
| 0 | 1.31E+03 | 8.36E+03 |
| 70 | 1.39E+03 | 8.23E+03 |
| 105 | 2.57E+03 | 1.47E+04 |
| 140 | 2.64E+03 | 1.46E+04 |
| 154 | 2.67E+03 | 1.46E+04 |
| 210 | 2.32E+03 | 1.21E+04 |
| 224 | 2.34E+03 | 1.20E+04 |
| 280 | 2.44E+03 | 1.19E+04 |

Supplementary Table 7 - Transfer of radon between the uterus and the placenta (d^-1^)

| Gestation age (days) | Uterus to placenta | Placenta to uterus |
| --- | --- | --- |
| 105 | 3.78E+03 | 5.57E+03 |
| 140 | 3.37E+03 | 4.56E+03 |
| 154 | 2.64E+03 | 3.84E+03 |
| 210 | 2.98E+03 | 2.67E+03 |
| 224 | 1.98E+03 | 2.55E+03 |
| 280 | 2.18E+03 | 2.26E+03 |

Supplementary Table 8 - Fetoplacental exchange of radon (d^-1^)

| Gestation age (days) | Placenta to V Cord | A Cord to placenta |
| --- | --- | --- |
| 105 | 4.77E+02 | 2.35E+03 |
| 140 | 4.67E+02 | 4.60E+03 |
| 154 | 4.23E+02 | 4.27E+03 |
| 210 | 6.03E+02 | 6.14E+03 |
| 224 | 6.29E+02 | 5.98E+03 |
| 280 | 6.98E+02 | 5.97E+03 |

Supplementary Table 9 - Transfer coefficients (d^-1^) from V Cord to fetal organs

| Gestation age (days) | Lungs | Brain | Kidneys | Bone | Liver | Adipose | Other | Thyroid | RBM |
| --- | --- | --- | --- | --- | --- | --- | --- | --- | --- |
| 105 | 1.73E+03 | 9.31E+02 | 6.84E+02 | 3.91E+02 | 1.59E+03 | 0.00E+00 | 0.00E+00 | 0.00E+00 | 0.00E+00 |
| 140 | 3.37E+03 | 2.11E+03 | 1.21E+03 | 7.66E+02 | 3.12E+03 | 0.00E+00 | 3.36E+02 | 2.54E-01 | 8.07E+00 |
| 154 | 3.13E+03 | 1.98E+03 | 1.12E+03 | 7.12E+02 | 2.90E+03 | 0.00E+00 | 3.28E+02 | 2.21E-01 | 7.11E+00 |
| 175 | 4.22E+03 | 2.93E+03 | 1.35E+03 | 1.07E+03 | 3.68E+03 | 9.58E+02 | 9.70E+02 | 5.59E-01 | 2.08E+01 |
| 210 | 4.50E+03 | 3.26E+03 | 1.37E+03 | 1.20E+03 | 3.82E+03 | 1.02E+03 | 1.30E+03 | 5.93E-01 | 2.53E+01 |
| 224 | 4.38E+03 | 3.20E+03 | 1.32E+03 | 1.18E+03 | 3.70E+03 | 9.95E+02 | 1.31E+03 | 5.37E-01 | 2.37E+01 |
| 280 | 4.38E+03 | 3.48E+03 | 1.12E+03 | 9.95E+02 | 3.30E+03 | 9.95E+02 | 2.50E+03 | 1.30E+00 | 4.69E+01 |

Supplementary Table 10 - Transfer coefficients (d^-1^) from fetal organs to A Cord

| Gestation age (days) | Lungs | Brain | Kidneys | Bone | Liver | Adipose | Other | Thyroid | RBM |
| --- | --- | --- | --- | --- | --- | --- | --- | --- | --- |
| 105 | 2.47E+03 | 2.93E+02 | 3.93E+03 | 9.90E+03 | 1.85E+03 | 0.00E+00 | 0.00E+00 | 0.00E+00 | 0.00E+00 |
| 140 | 5.90E+03 | 7.44E+02 | 7.15E+03 | 2.01E+04 | 3.88E+03 | 0.00E+00 | 1.11E+02 | 8.36E-02 | 2.66E+00 |
| 154 | 5.90E+03 | 7.13E+02 | 6.76E+03 | 1.91E+04 | 3.63E+03 | 0.00E+00 | 5.00E+01 | 3.38E-02 | 1.09E+00 |
| 175 | 8.39E+03 | 1.08E+03 | 8.32E+03 | 2.92E+04 | 4.64E+03 | 1.01E+02 | 1.49E+02 | 8.59E-02 | 3.20E+00 |
| 210 | 1.05E+04 | 1.25E+03 | 8.35E+03 | 3.35E+04 | 4.86E+03 | 4.09E+01 | 2.40E+02 | 1.09E-01 | 4.66E+00 |
| 224 | 1.10E+04 | 1.46E+03 | 8.23E+03 | 3.30E+04 | 4.72E+03 | 3.15E+01 | 1.80E+02 | 7.36E-02 | 3.25E+00 |
| 280 | 1.31E+04 | 1.50E+03 | 7.33E+03 | 2.79E+04 | 4.25E+03 | 2.24E+01 | 4.77E+02 | 2.48E-01 | 8.98E+00 |
